# HIV prevalence, spatial distribution and risk factors for HIV infection in the Kenyan fishing communities of Lake Victoria

**DOI:** 10.1371/journal.pone.0214360

**Published:** 2019-03-25

**Authors:** Zachary A. Kwena, Stella W. Njuguna, Ali Ssetala, Janet Seeley, Leslie Nielsen, Jan De Bont, Elizabeth A. Bukusi, Lake Victoria Consortium for Health Research (LVCHR) Team

**Affiliations:** 1 Kenya Medical Research Institute, Nairobi, Kenya; 2 UVRI-IAVI HIV Vaccine Program, Entebbe, Uganda; 3 MRC/UVRI Uganda Research Unit, Entebbe, Uganda; 4 International AIDS Vaccine Initiative, New York, United States of America; National Institute of Health, ITALY

## Abstract

**Introduction:**

Global efforts to end HIV by 2030 focus on reducing and eventually eliminating new infections in priority populations. Identifying these populations and characterizing their vulnerability factors helps in guiding investment of scarce HIV prevention resources to achieve maximum impact. We sought to establish HIV prevalence, spatial distribution and risk factors for HIV infection in the Kenyan fishing communities of Lake Victoria.

**Methods:**

We conducted a cross-sectional survey of 2637 people from all the 308 fish-landing beaches on the Kenyan shore of Lake Victoria. The number of participants enrolled at each beach were weighted based on the size of the beach, determined by the number of functional registered boats. We used simple random sampling to select those to be approached for study participation. Consenting participants were privately interviewed about their socio-economic and demographic characteristics and sexual behavior, and were invited for HIV test using the Kenya rapid HIV testing protocol. We used descriptive statistics and multivariate logistic and linear regression for analysis.

**Results:**

We found high HIV prevalence of 32% with significant differences between men (29%) and women (38%). Among men, having an HIV negative sexual partner, being circumcised, increasing number of condom protected sex acts in the preceding month, being younger and being a resident of Homa Bay, Kisumu, Siaya and Busia counties compared to Migori County reduced the risk of HIV infection. For women, being married, having more children with the current spouse, having an HIV negative sexual partner and being a resident of Busia compared to Migori County reduced the risk of HIV infection. We also found that longer distance from the beaches to the nearest public health facilities was associated with increasing cumulative HIV prevalence at the beaches.

**Conclusion:**

Fishing communities have high HIV prevalence and may greatly benefit from interventions such as wider ART coverage, couple HIV risk reduction counseling, PrEP use for HIV negative partner at substantial continuous risk, alongside other HIV prevention services that the Kenyan government is currently rolling out. This will additionally require adequate plans to synchronize the provision of these services with the population’s routine schedules for all these options to be reasonably accessible to them.

## Introduction

Achieving the UNAIDS goal of ending HIV by 2030 requires identifying HIV hotspots for targeted interventions to prevent new infections[1]. The current HIV prevention approaches advocate for geographic and subpopulation targeting in investing available resources for maximum impact[2]. Sub-Saharan Africa bears the greatest burden of the HIV epidemic described as generalized; but with substantial regional and subpopulation differences. The most affected are countries in southern and eastern Africa, such as Kenya, as well as occupational subpopulations of migrant workers, sex workers, long distance truck drivers and others like injection drug users (IDU) and men who have sex with men (MSM)[3–5]. These populations, characterized by high HIV prevalence may sometimes act as important sources of new HIV infections to the general population.

The UNAIDS 90-90-90 targets outline that at least 90% of population should be aware of their HIV status as an entry point into care that acts both as prevention and treatment[1]. This is more urgent in HIV hotspots to reduce transmission within the key populations and to the general population. To achieve these targets and the vision of an AIDS-free generation, it is essential to identify all most-at-risk subpopulations and provide services to increase awareness of their HIV status. Available literature from studies in small localized fishing communities from Kenya and Uganda show that Lake Victoria fishing communities, who comprise fishermen, fish traders/processors, boat owners and other traders selling assorted fishing commodities, as well as restaurant/bar workers and sex workers at the fish-landing beaches, are at a much higher risk of HIV infection compared to the general population[6–10]. HIV prevalence in the fish-landing beaches, defined as designated areas where fishing boats land with fish for sale, has been shown to range from anywhere between 12 to 32%[8, 11].

The high risk of HIV infection among fishing communities has been attributed to many factors that are both behavioral and structural in nature[12–16]. For instance, fisherfolk in many places, especially in sub-Saharan Africa including Kenya, are described as being highly mobile in pursuit of fish. As such, they are often away from homes and their families for long periods and interact a lot with women fish traders in the course of their work[17–20]. In the process of these interactions, fishermen end up forming casual sexual relationships known as *jaboya (fish-for-sex)* with women fish traders, which take place within the context of perpetual low condom use and high consumption of alcohol and drugs in the fishing villages[21–23]. Historically, fisherfolk on Lake Victoria tend to be socially marginalized and stigmatized, often with limited access to health and other social services. This is mainly because of the remote places with undeveloped road network and other infrastructure they live and work in. Health facilities are spatially inaccessible and the few within reach have working hours that are not favorable to fisherfolk[24, 25]. For instance, some fishermen leave for work offshore early in the morning and return in the late afternoon when most public health facilities are closed. Conversely, those who work offshore overnight return in the morning and spend most of their day sleeping; making them unavailable to access health services.

Given the prevailing social and contextual HIV risk environments in the fishing communities, it is important to obtain accurate estimates of the HIV prevalence in the Kenyan fishing communities and characterize their vulnerability to guide in the investment of scarce HIV prevention resources to achieve maximum impact in reducing new infections. Thus, we sought to establish HIV prevalence, spatial distribution and risk factors for HIV infection among men and women in the Kenyan fish-landing beaches on Lake Victoria.

## Methods

### Study design

We conducted a cross-sectional survey of a total of 2637 fisherfolk aged 18 to 76, randomly selected from all 308 Kenyan fish-landing beaches to establish HIV prevalence, spatial distribution and risk factors for HIV infection.

### Study site and population

The study area, located around the Kenyan shores of Lake Victoria, covers five counties of Migori, Homa Bay, Kisumu, Siaya and Busia. Other than parts of Busia County; the region is predominantly inhabited by the Luo community who traditionally do not practice male circumcision. Our study population consisted of fishermen, fish traders, boat makers/repairers, beach business people as well as other groups that included restaurant/bar workers and sex workers, residing and/or working in the fish-landing beaches[26].

### Study recruitment procedures

This study used existing organizational structures to enroll participants registered at the respective fish-landing beaches. Each fish-landing beach is managed by a Beach Management Unit (BMU) with elected officials. The BMUs keep registers of all fishing boats and the crew working on each. In addition, they also keep a record of all other people working at the beaches. We used the number of registered functional boats to estimate the size of the beach and determine the number of participants from our sample size to be recruited from each beach. This ensured that more participants were recruited from bigger beaches relative to smaller ones. To identify individual participants for interviews, we conducted a stratified random sampling; stratified by the occupational groups mentioned above. The number selected from each group to participate in the study was proportional to the number of people registered in each occupational group on each beach. The identified persons were approached, informed about the study and those interested referred to a specially pre-arranged venue close to the beaches where consenting and other study procedures were taking place.

### Data collection

This survey was conducted in November and December 2013 with our main outcome variable being HIV status. Arriving participants for the study were given written informed consent forms in one of the three languages commonly spoken in the study area, that is, English (official language), Kiswahili (national language) or Dholuo (local language); depending on which of the three they were comfortable with. They were then given sufficient time to read the consent forms before meeting a trained interviewer who went through the consent forms with them emphasizing key aspects of the consent form. Participants were allowed time to ask questions before interviewer confirmed their understanding of the consent form and asking them to sign if they were still interested in participating in the study. After consenting, every participant undergoing testing had blood collected by a finger stick for rapid HIV antibody testing. For each specimen, we used two test kits, Determine (Abbott Laboratories, Abbot Park, IL) and Bioline (Standard Diagnostics, Giheung-gu, Korea) approved by the Kenyan Ministry of Health. Determine was the first test used on all participants with Bioline only used on those testing positive on the first test. For discordant results, a third rapid test, Uni-gold (Trinity Biotech PLC, Bray, Ireland) was processed as a tiebreaker to determine the final result. Those testing positive were counseled and referred for further counseling, care and treatment. In addition to HIV status data, we also collected geo-spatial (GSP coordinates) as well as socio-demographic (gender, age, education, income), behavioral (alcohol consumption, multiple sexual partnerships, condom use) and mobility (number of beaches worked, months spent away from home) data. This study was approved KEMRI's Ethics Review Committee and adhered to international human subjects ethics standards.

### Data analysis

Data from CSPro was exported to SPSS version 21 for final cleaning and analysis. We used descriptive statistics to summarize socio-demographic and behavioral data. As a follow up to the descriptive analyses, we carried out a series of bivariate analyses testing the relationship between one independent variable at a time with our outcome variable of interest which was HIV infection. The explanatory variables were picked based on biological plausibility, clinical and intuitive relationship to outcome variable. This bivariate testing helped us in short listing variables for multivariate analysis at a cutoff significance level of p≤0.05. We then directly entered the shortlisted variables into the multivariate logistic model and reported the resultant adjusted odds ratios with their 95% confidence intervals. To establish distance between fish-landing beaches and nearest health facilities, we obtained Shapefiles Kenya administrative locations, health facilities, Lake Victoria which we plotted using QGIS software. We then overlaid the beach geocodes we collected during the survey. Using MMQGIS function within QGIS, we created hub line distances that helped us determine distance from each beach to the nearest public health facility. We used linear regression to establish the relationship between cumulative HIV prevalence in beaches, within an administrative location, and distances to the nearest public health facilities in those respective locations.

## Results

Table 1 shows socio-demographic and behavioral characteristics of enrolled participants. Overall, two thirds were male and the median age was 34 (IQR, 28–44). The majority (84%) were married, of whom 34% were in a polygynous relationship with a median of 3 children (IQR, 2–5). Overall, participants reported to earn a median monthly income of USD 90 (IQR, 60–150). In terms of occupation, half (51%) were fishermen; 28% identified themselves as fish traders, a majority (78%) of whom were women. The participants had been working in the fishing industry for a median of 8 years (IQR, 4–15). Half of the participants (49%) reported that they lived away from their spouse part of the year when traveling for work. Participants reporting sex in the preceding 30 days had a median of 3 (IQR, 0–6) occasions of unprotected sex acts. Of participants who knew their partners' HIV status, one quarter reported that their partners were HIV positive. One third of participants tested HIV positive during the study; 29% of men and 38% of women. Overall, 84% of participants who reported their partners to be HIV positive tested HIV positive themselves. There were significant differences between men and women in all socio-demographic and behavioral characteristics assessed apart from the place of recruitment (whether mainland or island) (p = 0.14), number of children with the current spouse (p = 0.06) and HIV positivity of participants with reported HIV positive partners (p = 0.48) (Table 1).

**Table 1:** Socio-demographic and behavioral characteristics of enrolled participants.

Characteristic (Categorical)	ALL		Male		Female		p-value (Chi-square)^#^
	N	%	N	%	N	%	
Gender	2637	100	1767	67	870	33	-
Marital status							
Married	2199	84	1554	88	645	75	<0.01
Separated	53	2	27	2	26	3	
Widowed	171	6	18	1	153	18	
Single (never married)	200	8	167	9	33	4	
Polygynous marriage	811	34	419	26	392	50	<0.01
Education							
Primary not completed	865	33	468	26	397	46	<0.01
Completed primary but not secondary	1182	45	811	46	371	43	
Completed secondary	574	22	485	28	89	11	
Occupation							
Fishing boatcrew	1339	51	1264	73	45	5	<0.01
Fish trader/agent	724	28	161	9	563	66	
Boat owner	138	5	111	6	27	3	
Business person	280	11	100	6	180	21	
Other occupations	141	5	99	6	42	5	
County of residence							
Migori	229	9	150	8	79	9	0.07
Homa Bay	1042	40	704	40	338	39	
Kisumu	232	9	139	8	93	11	
Siaya	790	30	552	31	238	28	
Busia	331	12	222	13	109	13	
Wealth index							
Poorest	519	20	307	17	212	25	<0.01
Poorer	529	20	317	18	212	25	
Middle	520	20	371	21	1499	17	
Richer	530	20	371	21	159	18	
Richest	526	20	401	23	125	15	
Place of recruitment							
Mainland	2214	84	1504	85	710	83	0.14
Island	410	16	263	15	147	17	
Circumcision status	794	46	794	46	-	-	-
Current contraceptive use	447	53	-	-	447	53	
Live away from spouse part of year	1162	49	842	53	320	41	<0.01
Number of sexual partners in preceding 6 months							
None	172	6	49	3	123	14	<0.01
One	1619	62	963	54	656	76	
Two	596	23	528	30	68	9	
Three and more	232	9	225	13	7	1	
Condom use with marital partners	497	28	356	29	141	27	0.25
Condom use with non-marital partners	245	56	168	56	77	55	0.48
Reported sexual partner's HIV positivity	409	24	207	21	139	32	<0.01
HIV positivity during survey	830	32	512	29	318	38	<0.01
HIV positivity of fisherfolk with reported HIV+partners	341	84	227	85	114	82	0.48
Characteristic (Continuous)	Median	IQR	Median	IQR	Median	IQR	p-value (t-test)^#^
Age	34	28–41	34	28–42	34	27–40	<0.01
Number of children with current spouse	4	2–5	4	2–6	4	2–5	0.06
Monthly income (US Dollars)	90	60–150	100	60–150	80	45–150	<0.01
Length of time working in fish-related activities (years)	8	4–15	10	5–15	5	2–10	<0.01
Age at first marriage	21	18–24	22	20–25	18	16–20	<0.01
Age at first sex	16	14–18	16	15–18	15	14–18	<0.01
Number of times had unprotected sex in preceding 30 days	3	0–6	3	0–6	2	0–4	<0.01

^#^Difference between male and female

The fish-landing beaches were a median of 2.6 kilometers (IQR 1.4–3.9) from the nearest public health facility. The beach with the longest distance to health facility was 11.7 kilometers away and accessing some facilities required crossing over either to the mainland or island. HIV prevalence was slightly lower in administrative locations in Busia and Kisumu counties compared to the other three counties of Migori, Homa Bay and Siaya.

Table 2 shows factors that were significantly associated with HIV infections at bivariate level. The factors that were significant for combined data (both men and women) were: gender, age, level of education, wealth index, marital status, number of children with current spouse, county of residence, place of recruitment (whether mainland or island) and sexual partner's HIV status. For men, the significant factors were: age, level of education, marital status, county of residence, place of recruitment, sexual partner's HIV status and, circumcision status. For women, the significant factors were: wealth index, marital status, number of children with current spouse, county of residence, involvement in transactional sex and, main sexual partner's HIV status (Table 2).

**Table 2:** Unadjusted factors associated with HIV infection among fisherfolk in the Kenyan fishing communities on Lake Victoria.

Attribute	All	p-value	Male	p-value	Female	p-value
	OR(95%CI)		OR(95%CI)		OR(95%CI)	
Gender						
Male	1.00					
Female	1.43(1.21–1.71)	<0.01	N/A		N/A	
Age	1.01(1.01–1.02)	<0.01	1.02(1.01–1.03)	<0.01	1.00(0.99–1.01)	0.80
Education level						
Primary not completed	1.00					
Completed primary not secondary	0.86(0.71–1.04)	0.11	0.87(0.68–1.11)	0.27	0.93(0.69–1.24)	0.62
Completed secondary	0.67(0.53–0.85)	< 0.01	0.68(0.51–0.90)	0.01	0.95(0.59–1.54)	0.85
Wealth index						
Poorest	1.00					
Poorer	1.09(0.83–1.42)	0.53	0.98(0.69–1.40)	0.93	1.26(0.84–1.88)	0.27
Middle	1.49(1.15–1.93)	< 0.01	1.36(0.97–1.89)	0.07	1.99(1.29–3.06)	<0.01
Richer	1.09(0.84–1.42)	0.52	1.03(0.73–1.44)	0.87	1.31(0.85–2.03)	0.22
Richest	1.10(0.84–1.43)	0.49	1.09(0.78–1.52)	0.62	1.27(0.80–2.02)	0.31
Marital status						
Separated/widowed	1.00					
Married	0.37(0.28–0.49)	<0.01	1.03(0.54–1.95)	0.94	0.28(0.20–0.40)	<0.01
Single(never married)	0.10(0.06–0.17)	<0.01	0.20(0.09–0.47)	<0.01	0.21(0.09–0.49)	<0.01
Number of children with current spouse	0.92(0.89–0.95)	<0.01	0.99(0.94–1.03)	0.50	0.79(0.74–0.85)	<0.01
County of recruitment						
Migori	1.00					
HomaBay	0.93(0.69–1.26)	0.66	1.03(0.70–1.51)	0.87	0.81(0.49–1.33)	0.40
Kisumu	0.61(0.41–0.91)	0.01	0.63(0.37–0.06)	0.08	0.54(0.29–1.00)	0.05
Siaya	0.75(0.55–1.02)	0.06	0.81(0.55–1.20)	0.29	0.68(0.41–1.15)	0.15
Busia	0.26(0.17–0.40)	<0.01	0.33(0.20–0.55)	<0.01	0.17(0.09–0.35)	<0.01
Place recruited						
Mainland	1.00					
Island	1.42(1.14–1.78)	<0.01	1.42(1.07–1.88)	0.01	1.38(0.96–1.98)	0.08
Involvement in transactional sex						
No	1.00					
Yes	1.02(0.82–1.26)	0.86	0.84(0.65–1.09)	0.19	1.90(1.26–2.85)	<0.01
Reported sexual partner's HIV status						
Positive	1.00					
Negative	0.04(0.03–0.05)	<0.01	0.04(0.02–0.05)	<0.01	0.05(0.03–0.09)	<0.01
Circumcision status						
Not circumcised	1.00					
Circumcised	N/A	-	0.61(0.49–0.75)	<0.01	N/A	-

Table 3 shows results of multivariate analysis of factors that were significant at bivariate level. For combined data, the only factor that was independently associated with increased odds of HIV infection was increasing age (adjusted odds ratio [aOR], 1.03; 95% confidence interval [95%CI] 1.01–1.04). Those that were associated with reduced odds of HIV infections were: being in the poorer wealth index category (aOR, 0.51; 95%CI 0.32–0.80), being in the richest wealth index category (aOR, 0.62; 95%CI 0.40–0.96), every one additional child with current spouse (aOR, 0.89; 95%CI 0.83–0.95), being married (aOR, 0.35; 95%CI 0.18–0.68), county of residence being Kisumu (aOR, 0.48; 95%CI 0.25–0.90) or Busia (aOR, 0.21; 95%CI 0.11–0.39). We also found that reported main sexual partner's HIV status being negative (aOR, 0.04; 95%CI 0.03–0.06) was associated with reduced odds of HIV infection.

**Table 3:** Factors associated with HIV infection among fisherfolk in the Kenyan fishing communities on Lake Victoria.

Attribute	All		Male		Female	
	aOR(95%CI)	p-value	aOR(95%CI)	p-value	aOR(95%CI)	p-value
Gender						
Male	1.00					
Female	0.92(0.66–1.27)	0.60	N/A	N/A	N/A	-
Age	1.03(1.01–1.04)	<0.01	1.01(0.99–1.03)	0.14	N/A	-
Education level						
Primary not completed	1.00					
Completed primary not secondary	1.06(0.77–1.45)	0.73	0.95(0.64–1.40)	0.81	N/A	-
Completed secondary	0.88(0.59–1.31)	0.53	0.80(0.51–1.24)	0.31	N/A	-
Wealth index						
Poorest	1.00					
Poorer	0.51(0.32–0.80)	<0.01	N/A	-	0.50(0.21–1.17)	0.11
Middle	0.79(0.52–1.22)	0.29	N/A	-	0.49(0.20–1.19)	0.11
Richer	0.69(0.45–1.07)	0.10	N/A	-	0.76(0.33–1.77)	0.53
Richest	0.62(0.40–0 .96)	0.03	N/A	-	0.75(0.30–1.87)	0.54
Marital status						
Separated/widowed	1.00					
Married	0.35(0.18–0.68)	<0.01	1.70(0.43–6.66)	0.45	0.14(0.06–0.36)	<0.01
Single(never married)	0.39(0.10–1.50)	0.17	0.75(0.14–3.99)	0.73	0.11(0.01–0.97)	0.05
Number of children with current spouse	0.89(0.83–0.95)	<0.01	N/A	-	0.74(0.64–0.86)	<0.01
County of recruitment						
Migori	1.00					
Homa Bay	0.66(0.41–1.06)	0.09	0.60(0.34–1.03)	0.06	0.78(0.29–2.10)	0.62
Kisumu	0.48(0.25–0.90)	0.02	0.34(0.15–0.76)	0.01	0.77(0.22–2.68)	0.68
Siaya	0.63(0.39–1.03)	0.06	0.60(0.34–1.05)	0.07	71(0.25–1.98)	0.51
Busia	0.21(0.11–0.39)	<0.01	0.26(0.13–0.55)	<0.01	0.23(0.06–0.88)	0.03
Place recruited						
Mainland	1.00					
Island	1.27(0.87–1.84)	0.22	1.42(0.93–2.19)	0.10	N/A	-
Involvement in transactional sex						
No	1.00					
Yes	N/A		N/A	-	0.79(0.35–1.79)	0.58
Reported sexual partner's HIV status						
Positive	1.00					
Negative	0.04(0.03–0.06)	<0.01	0.04(0.03–0.06)	<0.01	0.04(0.02–0.08)	<0.01
Circumcision status						
Not circumcised	1.00					
Circumcised	N/A	-	0.65(0.47–0.91)	<0.01	N/A	-

Among men, factors that were independently associated with reduced odds of HIV infections were: county of residence being Kisumu (aOR, 0.34; 95%CI 0.15–0.76) or Busia (aOR, 0.26; 95%CI 0.13–0.55), reported main sexual partner's HIV status being negative (aOR, 0.04; 95%CI 0.03–0.06) and being circumcised (aOR, 0.65 95%CI 0.47–0.91) (Table 3). For women, the factors that were independently associated with reduced odds of HIV infections were: being married (aOR, 0.14; 95%CI 0.06–0.36), being single (never married) (aOR, 0.11; 95%CI 0.01–0.97), increasing number of children with current spouse (aOR, 0.74; 95%CI 0.64–0.86), county of residence being Busia (aOR, 0.23; 95%CI 0.06–0.88) and, reported main sexual partner's HIV status being negative (aOR, 0.04; 95%CI 0.02–0.08) (Table 3). Spatial distance to the nearest public health facilities at the beach was associated with increasing cumulative HIV prevalence at the beaches in the administrative locations. One kilometer longer distance to nearest public health facility was associated with 17% higher HIV prevalence at the beaches in the locations.

## Discussion

This was one of the first cross-sectional survey conducting HIV testing and at the same time obtaining geographic location of all fish-landing beaches on the Kenyan side of Lake Victoria with a goal of establishing the prevalence of HIV and the risk factors for infection. We found high HIV prevalence of 32% with significantly higher prevalence among women (38%) compared to men (29%). Overall, reduced odds of HIV infection was independently associated with being younger in age, being in the poorer and in the richest wealth index category compared to poorest, increasing number of children with current spouse, being currently married compared to being widowed or separated, county of residence being Kisumu or Busia compared to Migori and, reporting main sexual partner's HIV status being negative. The level of education, length of time away from spouse and involvement in transactional sex were not significantly associated with HIV infection.

Identifying HIV hotspots such as these fishing communities is one of the significant gaps that remain that are crucial to reaching the UNAIDS 90-90-90 targets by 2020 and ending HIV by 2030. To achieve 90-90-90 goal in Kenya, for instance, an additional 500,000 people living with HIV (PLHIV) need to be identified and initiated on ART, leading to over 400,000 PLHIV achieving viral load suppression[27]. Thus, with Kenyan fishing communities identified as one of the HIV hotspots, it is critically essential to put in place mechanisms to control new infections[28]. As Rousseau[29] argues, the basis for controlling new infections is understanding a population's structural as well as socio-economic vulnerabilities to develop tailor-made HIV prevention responses instead of applying a standard template everywhere and on everyone. Many sub-Saharan countries, including Kenya, are in the process of mapping out HIV hotspots to help in zooming in and appropriately directing available resources for maximum impact[30]. Additionally, identifying and zooming into hotspots helps to reveal whether certain geographic areas or key populations are being missed, neglected or inadequately serviced[31]. In fact, a modeling based on Kenya’s HIV epidemic clearly shows that tailoring interventions such as VMMC and couples' PrEP to the various specific patterns of HIV risk across hotspots has the potential to prevent up to 600,000 HIV infections by 2030 on the same budget[32].

Our findings show that HIV transmission within marriage is a major issue that needs to be urgently addressed as a HIV prevention strategy. This is because over 84% of men and women who reported their sexual partners to be HIV positive tested positive for HIV. These findings are consistent with data from other studies in the same region that show HIV serodiscordance of between 15–17%[8, 33]. This is especially true in the context of low marital and even extramarital condom use that is common in the fishing communities[23, 34]. For instance, a study enrolling married couples to determine the prevalence of HIV and correlates of HIV infection found that 17% of the partners within a couple were serodiscordant[8]. Currently, there exist various biomedical as well as socio-behavioral prevention options such as treatment as prevention, PrEP and, couple counseling that can benefit couples at risk of HIV infections. For instance, despite challenges in getting men to accompany their spouses to receive counseling together[35], couple counseling in the context of HIV testing has shown tremendous results in promoting safe sex within and even outside marital unions[36, 37]. A Zambian study among couples demonstrated substantial and sustained longitudinal reductions in self-reported unprotected sex after couple HIV counseling and testing[37].

The benefits of rapid ART scale-up and wider coverage that gave rise to undetectable = untransmittable (U = U) campaigns cannot be emphasized enough especially in serodiscordant relationships[38]. A paper presented at IAS 2018 conference by Rodger and colleagues delivered the last piece of evidence to validate U = U campaigns[38]. However, these benefits may not be available in areas where ART coverage and, more importantly, retention and adherence to medication at individual level cannot be achieved. Although we did not measure directly ART coverage in the Kenyan fishing communities where this study was conducted, a recent study from the neighboring Ugandan fishing communities show low uptake of 13% in men and 18% in women compared to trading and agrarian communities[39]. According to Kenya HIV county profile report, average ART coverage in the general population in counties that border Lake Victoria is reported to be in the nineties[40]. Despite this seemingly impressive coverage, the challenge has always been retention in care, especially in highly mobile fishing communities[41–43]. Thus, with limited of data in ART coverage in these fishing communities and potential of poor retention in HIV, it is important to emphasize use of the other HIV prevention options such as PrEP alongside continued scale up of ART coverage as well as retention in care programs.

Use of PrEP could also be an alternative intervention to couples and other individuals at high risk to prevent HIV transmission. PrEP that has been shown to dramatically reduce the risk of HIV acquisition[44, 45] and currently being rolled out in Kenya, holds potential to benefit individuals who are at a continuous heightened risk of HIV infections such as those in serodiscordant relationships including HIV negative but at risk of infection[46]. Public health challenge for serodiscordant couples, in settings such as fishing communities with serodiscordance rate as high as 17%[8], is how to prevent the HIV negative individuals from serocoverting. Using combined delivery of antiretroviral therapy (ART) for HIV-positive partners and time-limited PrEP for negative partners can virtually eliminate HIV transmission to the negative partner[47]. Despite reservations about the circumstances of combined PrEP and ART use[48], Ying et al. show, through mathematical modeling, that using PrEP and ART for high-risk persons have the potential for synergistic action and are cost-effective in preventing HIV infections in high prevalence settings[49]. This needs substantial counseling of couples and even individuals coupled with providing information about HIV serodiscordancy as they work to have the infected partner on ART and suppressed.

Even though PrEP may seem like an ideal prevention strategy for this population with low condom use, its effectiveness largely depends on whether it is rolled out in a way that recognizes and deliberately plans for how to overcome the traditional health access barriers in the fishing communities. The major access barriers to health services in the fishing communities have been identified as distance to health public health facilities and unsynchronized and uncoordinated timings between the fisherfolk's availability and the opening and closing hours of the health facilities[24]. These barriers partly explain the suboptimal uptake of many promising interventions such as ART and VMMC[50] which initially received overwhelming political and financial support. Although we have had some substantial increase in both ART and VMMC coverage in the recent past, retention in ART programs among adolescents and young people as well as VMMC coverage of older men aged above 24 has been a challenge[51–53]. With fishing industry attracting relatively young people (with an average age of about 35) coupled with challenges of health service provision in the fishing communities[54], ART and VMMC coverage may be much lower than in the general population such as in Gem sub-county of Siaya County where Bordorff and colleagues conducted their surveys that showed a drop in HIV incidences relative to ART and VMMC coverage[55].

Indeed, our findings show that longer distance to nearest public health facilities was associated with increase in cumulative HIV prevalence at the beaches. Although intervening factors may not be obvious, it is possible that the relationship could be as a result of challenges in accessing HIV prevention services such as condoms, STI screening and, HIV testing and enrolment and retention in care for those diagnosed with HIV. While HIV testing empowers people to engage in safe sex and is an entry point to HIV care[56–58], failure to initiate care in a timely manner compromises viral suppression and reduction of infectiousness[59]. This perpetuates HIV circulation within the community resulting in high prevalence. Remote locations, high mobility and general neglect by governments have hampered fishing communities' access to health services[24]. Several other studies have similarly shown that distance to health facilities hampers access to health services[60, 61].

The Kenyan side of Lake Victoria has five counties; four of which (Migori, Homa Bay, Kisumu, Siaya) are inhabited by the Luo community who are traditionally non-circumcising while Busia is inhabited by the traditionally circumcising Luhya community. We have shown that the county of residence was an important factor in HIV infection. The ethnic composition of these counties and its relationship with circumcision may explain the differences in HIV prevalence between beaches in Migori (39%) Homa Bay (37%), Siaya (32%) and Kisumu (28%) counties compared to those in Busia County (14%). While ethnicity may not be a modifiable factor that can inform intervention design, it nonetheless points to the areas where we need to invest more resources as well as offering lessons about cultural differences that might be important in HIV prevention. Interestingly, despite the government's efforts to scale up VMMC in the four counties predominantly inhabited by the Luo community, the coverage in the fishing communities is still low at 46% compared to the regional coverage of 71% and desired program target of 80%[62, 63]. Recent studies conducted by Akullian and colleagues in 2014 and Odoyo-June and colleagues in 2015 show less than five percentage point increase in VMMC coverage in the general population in Nyanza region compared to the data we obtained in this study[64, 65]. Even then, the larger part of this increase was accounted for by in- school youth who are thought to be relatively easy to mobilize and are likely to accept the service. It is likely that due to infrastructural and logistical challenges of providing health services in the fishing communities, the percent increase would be much smaller had they targeted men in these communities.

Our findings show that people reporting being widowed or separated had higher odds of being HIV infected compared to those in marriage. This is consistent with other findings elsewhere in sub-Saharan Africa[66, 67]. For instance, Tenkorang using data from Demographic and Health Surveys combined with AIDS Indicator Survey from seven East and South African countries show that widowed people, especially women, are at a much higher risk of infection compared to married or never-married. Their vulnerability to HIV infection could be due to cultural rituals surrounding death of a spouse in many parts of sub-Saharan Africa as well as the high probability that a widow’s spouse may have died of AIDS[68, 69]. This is because many people are unaware of their own and/or even their partner’s HIV status hence they may have been exposed without knowing.

This study had several limitations. This study was conducted in 2013 and there is a possibility that, due to rapidly advancing HIV field in terms of ART and VMMC coverage, HIV prevalence and even the risk factors could have changed and may not accurately represent the current scenario if we go by the lessons learnt from VMMC and ART scale-up in rural Kwazulu-Natal, South Africa[70] and closer home in Gem sub-County[55]. However, we believe the rate of VMMC and ART coverage in the fishing communities may be lower than in the general population in rural Kwazulu-Natal and Gem because of challenges in health service provision in fishing communities. Thus, we believe these data is still worth sharing to consciously inform current HIV intervention efforts, especially in the fishing communities that often receive less attention. Given our overall study sample size, we selected the number of fisherfolk to be enrolled in each beach proportional to the size of the beach. Thus, in some beaches and even administrative locations in the counties, the numbers enrolled were so small to be representative of the fisherfolk at the beaches or administrative locations. Although we ensured that selection of participants was conducted in a random manner, we could not completely rule out selection biases among those who accepted to participate relative to the few who declined. This study was partly designed to retrospectively collect information about participants, some of which was sensitive sexual activities including—extra-marital partnerships. Thus, it was not possible to completely eliminate social desirability biases associated with self-report as well as recall biases. It is conceivable that fisherfolk in these prevailing contexts might have under-reported certain behaviors to conform to community norms[71]. Despite the reassurances about confidentiality we gave to our participants, some might still have been uncomfortable discussing sensitive information with us for fear of it leaking back to their communities. This fear might have inadvertently exacerbated the social desirability biases in responding to sensitive questions. As inherent in all cross-sectional studies, we were limited to establishing existence of relationships between variables but not causality. For example, we could not definitively tell whether circumcision that is associated with HIV infection in this study occurred before or after HIV infection which can only be establish longitudinal studies. These limitations notwithstanding, the study contributes to identifying fishing communities as HIV priority populations that need tailored interventions to shut down new infections. This is in support of UNAIDS targets of achieving 90-90-90 by 2020 and the vision of ending HIV epidemic by 2030.

## Conclusion

In conclusion, we found high HIV prevalence of 32% with gender differences in terms of HIV prevalence and associated risk factors. Overall, HIV infections seemed to more frequently occur in beaches with poor access to health services due to distance and among serodiscordant marital and stable partnerships. Fishing communities can greatly benefit from wide ART coverage that aim at achieving high retention and viral suppression in addition to targeted prevention efforts such as couple risk-reduction counseling and PrEP roll out. However, there is need to ensure that deliberate plans are put in place to synchronize the provision of these services with fisherfolk's availability.

## Supporting information

S1 Dataset Dataset used to generate results presented. (SAV)

## References

[1] UNAIDS. 90-90-90: an ambitious treatment target to help end the AIDS epidemic Geneve: UNAIDS 2014.

[2] Lakew Y, Benedict S, Haile D. Social determinants of HIV infection, hotspot areas and subpopulation groups in Ethiopia: evidence from the National Demographic and Health Survey in 2011. BMJ Open. 2015,5:e008669 10.1136/bmjopen-2015-008669 26589427 PMC4663400

[3] Sanders EJ, Okuku HS, Smith AD, Mwangome M, Wahome E, Fegan G, et al High HIV-1 incidence, correlates of HIV-1 acquisition, and high viral loads following seroconversion among MSM. AIDS. 2013,27:437–446. 10.1097/QAD.0b013e32835b0f81 23079811 PMC3929859

[4] Jackson DJ, Rakwar JP, Richardson BA, Mandaliya K, Chohan BH, Bwayo JJ, et al Decreased incidence of sexually transmitted diseases among trucking company workers in Kenya: results of a behavioural risk-reduction programme. AIDS. 1997,11. 10.1097/00002030-199707000-00010 9189216

[5] Baral S, Beyrer C, Muessig K, Poteat T, Wirtz AL, Decker MR, et al Burden of HIV among female sex workers in low-income and middle-income countries: a systematic review and meta-analysis. Lancet Infect Dis. 2012,12:538–549. 10.1016/S1473-3099(12)70066-X 22424777

[6] Ondondo RO, Waithera Ng’ang’a Z, Mpoke S, Kiptoo M, Bukusi EA. Prevalence and incidence of HIV infection among fishermen along Lake Victoria beaches in Kisumu County, Kenya. World J AIDS. 2014,4:219.

[7] Kiwanuka N, Ssetaala A, Nalutaaya A, Mpendo J, Wambuzi M, Nanvubya A, et al High incidence of HIV-1 infection in a general population of fishing communities around Lake Victoria, Uganda. PLoS ONE. 2014,9:e94932 10.1371/journal.pone.0094932 24866840 PMC4035272

[8] Kwena ZA, Ang'awa D, Bukusi EA. HIV prevalence, incidence and possible sources of infections among married couples in the fishing communities in Western Kenya In: Center for AIDS Research Sub‐Saharan Africa Working Group (CFAR‐SSA) Durban; 2016.

[9] Kiwanuka N, Ssetaala A, Mpendo J, Wambuzi M, Nanvubya A, Sigirenda S, et al High HIV-1 prevalence, risk behaviours, and willingness to participate in HIV vaccine trials in fishing communities on Lake Victoria, Uganda. JInt AIDS Soc 2013,16. 10.7448/IAS.16.1.18621 PMC3720985 23880102

[10] Opio A, Muyonga M, Mulumba N. HIV infection in fishing communities of Lake Victoria basin of Uganda: a cross-sectional sero-behavioral survey. PloS ONE. 2013,8:e70770 10.1371/journal.pone.0070770 23940638 PMC3733779

[11] Kwena Z, Njuguna S, Ondondo R, Njoroge B, Bukusi E. Regional HIV prevalence and associated risk factors among fisherfolk on the shores of Lake Victoria, Kenya. In: 7th Semiannual East Africa Collaborative Scientific Symposium. Jumuia Hotel Kisumu; 2014.

[12] Kwena ZA, Mwanzo IJ, Bukusi EA, Achiro LF, Shisanya CA. Across-sectional survey of prevalence and correlates of couple sexual concurrency among married couples in fishing communities along Lake Victoria in Kisumu, Kenya. Sex Transm Infect. 2013. 10.1136/sextrans-2013-051168 PMC5608652 24154655

[13] Asiki G, Mpendo J, Abaasa A, Agaba C, Nanvubya A, Nielsen L, et al HIV and syphilis prevalence and associated risk factors among fishing communities of Lake Victoria, Uganda. Sex Transm Infect. 2011,87:511–515. 10.1136/sti.2010.046805 21835763

[14] Lubega M, Nakyaanjo N, Nansubuga S, Hiire E, Kigozi G, Nakigozi G, et al Risk denial and socio-economic factors related to high HIV transmission in a fishing community in Rakai, Uganda: a qualitative study. PLoS ONE. 2015,10:e0132740 10.1371/journal.pone.0132740 26309179 PMC4550390

[15] Lubega M, Nakyaanjo N, Nansubuga S, Hiire E, Kigozi G, Nakigozi G, et al Understanding the socio-structural context of high HIV transmission in Kasensero fishing community, southwestern Uganda. BMC Public Health. 2015,15:1033 10.1186/s12889-015-2371-4 26449622 PMC4599692

[16] Seeley J, Nakiyingi-Miiro J, Kamali A, Mpendo J, Asiki G, Abaasa A, et al High HIV incidence and socio-behavioral risk patterns in fishing communities on the shores of Lake Victoria, Uganda. Sex Transm Dis. 2012,39:433–439. 10.1097/OLQ.0b013e318251555d 22592828

[17] Kwena ZA, Cohen CR, Sang NM, Ng'ayo MO, Ochieng JH, Bukusi EA. Fishermen as a suitable population for HIV intervention trials. AIDS Res Treat.2010:865903 10.1155/2010/865903 21490906 PMC3065805

[18] Nagoli J, Holvoet K, Remme M. HIV and AIDS vulnerability in fishing communities in Mangochi district, Malawi. Afr J AIDS Res. 2010,9:71–80. 10.2989/16085906.2010.484575 25860415

[19] Kissling E, Allison EH, Seeley JA, Russell S, Bachmann M, Musgrave SD, et al Fisherfolk are among groups most at risk of HIV: cross-country analysis of prevalence and numbers infected. AIDS (London, England). 2005,19:1939–1946. 10.1097/01.aids.0000191925.54679.94 16260899

[20] Allison EA, Seeley JA. HIV and AIDS among fisherfolk: a threat to ‘responsible fisheries'? 2004,5:215–234.

[21] Kwena ZA, Bukusi E, Omondi E, Ng'ayo M, Holmes KK. Transactional sex in the fishing communities along Lake Victoria, Kenya: a catalyst for the spread of HIV. Afr J AIDS Res 2012,11:9–15. 10.2989/16085906.2012.671267 25870893

[22] Kwena ZA, Bukusi EA, Ng'ayo MO, Buffardi AL, Nguti R, Richardson B, et al Prevalence and risk factors for sexually transmitted infections in a high-risk occupational group: the case of fishermen along Lake Victoria in Kisumu, Kenya. Int J STD AIDS 2010,21:708–713. 10.1258/ijsa.2010.010160 21139150

[23] Tumwesigye NM, Atuyambe L, Wanyenze RK, Kibira SP, Li Q, Wabwire-Mangen F, et al Alcohol consumption and risky sexual behaviour in the fishing communities: evidence from two fish landing sites on Lake Victoria in Uganda. BMC public health 2012,12:1069 10.1186/1471-2458-12-1069 23231779 PMC3534008

[24] Kwena ZA. HIV in fishing communities: contexts, prevalence, incidence, risk factors and interventions In: CROI Boston, USA; 2016.

[25] Seeley JA, Allison EH. HIV/AIDS in fishing communities: challenges to delivering antiretroviral therapy to vulnerable groups. AIDS care 2005,17:688–697. 10.1080/09540120412331336698 16036255

[26] Government of Kenya. Fisheries annual statistical bulletin In. Nairobi: Government printers; 2006.

[27] NASCOP. National plan for accelerating HIV care and treatment, 2015–2017. In. Nairobi; 2015.

[28] Kharsany AB, Karim QA. HIV Infection and AIDS in Sub-Saharan Africa: Current Status, Challenges and Opportunities. Open AIDS J 2016,10:34–48. 10.2174/1874613601610010034 27347270 PMC4893541

[29] Rousseau P. HIV hotspot mapping: an evidence-informed HIV response. In; 2016.

[30] UNAIDS. Local epidemics issues brief. In. Geneva; 2014.

[31] Cuadros DF, Awad SF, Abu-Raddad LJ. Mapping HIV clustering: a strategy for identifying populations at high risk ofHIV infection in sub-Saharan Africa. Int J Health Geogr 2013,12:28 10.1186/1476-072X-12-28 23692994 PMC3669110

[32] Anderson SJ, Cherutich P, Kilonzo N, Cremin I, Fecht D, Kimanga D, Harper M, Masha RL, Ngongo PB, Maina W, Dybul M, Hallett TB. Maximising the effect of combination HIV prevention through prioritisation of the people and places in greatest need: a modelling study. Lancet. 2014 7 19;384(9939):249–56. 10.1016/S0140-6736(14)61053-9 25042235

[33] Yang RR, Gui X, Xiong Y, Gao SC, Yan YJ. Five-year follow-up observation of HIV prevalence in serodiscordant couples. Int J Infect Dis. 2015;33:179–84. 10.1016/j.ijid.2015.02.007 25677723

[34] Kwena Z, Ang'awa D, Makokha C, Bukusi EA. High risk behavior in married people living with HIV: implications for prevention. In: Conference on Retroviruses and Opportunistic Infections (CROI). Washington State Convention Center, Seattle, USA.; 2017.

[35] Matovu JK, Wanyenze RK, Wabwire-Mangen F, Nakubulwa R, Sekamwa R, Masika A, et al "Men are always scared to test with their partners … it is like taking them to the Police": Motivations for and barriers to couples' HIV counselling and testing in Rakai, Uganda: a qualitative study. J Int AIDS Soc; 2014;17:19160 10.7448/IAS.17.1.19160 25239379 PMC4169647

[36] Jones D, Kashy D, Chitalu N, Kankasa C, Mumbi M, Cook R, et al Risk reduction among HIV-seroconcordant and -discordant couples: the Zambia NOW2 intervention. AIDS Patient Care STDS; 2014;28:433–441. 10.1089/apc.2014.0039 24983201 PMC4117267

[37] Wall KM, Kilembe W, Vwalika B, Haddad LB, Lakhi S, Onwubiko U, et al Sustained effect of couples' HIV counselling and testing on risk reduction among Zambian HIV serodiscordant couples. Sex Transm Infect. 2017;93:259–266. 10.1136/sextrans-2016-052743 28082662 PMC5520263

[38] Rodger A., Cambiano V, Bruun T, Vernazza P, Collins S, Corbelli GM, Degen O, Estrada V, Geretti AM, Beloukas A, Phillips AN, Lundgren J, for the PARTNER Study Group. Risk of HIV transmission through condomless sex in MSM couples with suppressive ART: The PARTNER2 Study extended results in gay men In AIDS 2018 Conference. RAI Convention Center, Amsterdam, The Netherlands; 2018

[39] Chang LW, Grabowski MK, Ssekubugu R, Nalugoda F, Kigozi G, Nantume B, Lessler J, Moore SM, Quinn TC, Reynolds SJ, Gray RH, Serwadda D, Wawer MJ. Heterogeneity of the HIV epidemic in agrarian, trading, and fishing communities in Rakai, Uganda: an observational epidemiological study. Lancet HIV 2016; 3: e388–96 10.1016/S2352-3018(16)30034-0 27470029 PMC4973864

[40] NACC NASCOP. Kenya HIV county profiles. In. Nairobi: Government printers; 2016.

[41] Bogart LM, Naigino R, Maistrellis E, Wagner GJ, Musoke W, Mukasa B, Jumamil R, Wanyenze RK. Barriers to Linkage to HIV Care in Ugandan Fisherfolk Communities: A Qualitative Analysis. AIDS Behav. 2016;20:2464–2476. 10.1007/s10461-016-1331-z 26961380 PMC5507699

[42] Geng EH, Glidden DV, Bwana MB, Musinguzi N, Emenyonu N, Muyindike W, Christopoulos KA, Neilands TB, Yiannoutsos CT, Deeks SG, Bangsberg DR, Martin JN. Retention in care and connection to care among HIV-infected patients on antiretroviral therapy in Africa: estimation via a sampling-based approach. PLoS ONE. 2011;6:e21797 10.1371/journal.pone.0021797 21818265 PMC3144217

[43] Bulsara SM, Wainberg ML, Newton-John TRO. Predictors of Adult Retention in HIV Care: A Systematic Review. AIDS Behav. 2018 3;22(3):752–764. 10.1007/s10461-016-1644-y 27990582 PMC5476508

[44] Baeten JM, Donnell D, Ndase P, Mugo NR, Campbell JD, Wangisi J, et al Antiretroviral prophylaxis for HIV prevention in heterosexual men and women. N Engl JMed. 2012;367:399–410. 22784037 10.1056/NEJMoa1108524 PMC3770474

[45] McCormack S, Dunn DT, Desai M, Dolling DI, Gafos M, Gilson R, et al Pre-exposure prophylaxis to prevent the acquisition of HIV-1 infection (PROUD): effectiveness results from the pilot phase of a pragmatic open-label randomised trial. Lancet. 2016;387:53–60. 10.1016/S0140-6736(15)00056-2 26364263 PMC4700047

[46] Namey E, Agot K, Ahmed K, Odhiambo J, Skhosana J, Guest G, et al When and why women might suspend PrEP use according to perceived seasons of risk: implications for PrEP-specific risk-reduction counselling. Cult Health Sex. 2016;18:1081–1091. 10.1080/13691058.2016.1164899 27093238 PMC5049692

[47] Morton JF, Celum C, Njoroge J, Nakyanzi A, Wakhungu I, Tindimwebwa E, et al Counseling Framework for HIV-Serodiscordant Couples on the Integrated Use of Antiretroviral Therapy and Pre-exposure Prophylaxis for HIV Prevention. J Acquir Immune Defic Syndr. 2017;74 Suppl 1:S15–S22. 27930607 10.1097/QAI.0000000000001210 PMC5147040

[48] Celum C, Hallett TB, Baeten JM. HIV-1 prevention with ART and PrEP: mathematical modeling insights into resistance, effectiveness, and public health impact. J Infect Dis. 2013;208:189–191. 10.1093/infdis/jit154 23570851 PMC3685227

[49] Ying R, Sharma M, Heffron R, Celum CL, Baeten JM, Katabira E, et al Cost-effectiveness of pre-exposure prophylaxis targeted to high-risk serodiscordant couples as a bridge to sustained ART use in Kampala, Uganda. J Int AIDS Soc. 2015;18:20013 10.7448/IAS.18.4.20013 26198348 PMC4509901

[50] Kwena ZA, Njuguna S, Njoroge B, Ondondo R, Muga C, Bukusi EA. Circumcision coverage, predictors of uptake and association with HIV infection in the fishing communities on Lake Victoria, Kenya In: University of Nairobi Annual Collaborative Meeting. Hotel Southern Sun Mayfair, Nairobi; 2015.

[51] Izudi J, Mugenyi J, Mugabekazi M, Muwanika B, Tumukunde Spector V, Katawera A, Kekitiinwa A. Retention of HIV-positive adolescents in care: a quality improvement intervention in mid-western Uganda. Biomed Res Int. 2018;6;2018:1524016 10.1155/2018/1524016 29854727 PMC5960514

[52] Okoboi S, Ssali L, Yansaneh AI, Bakanda C, Birungi J, Nantume S, Okullu JL, Sharp AR, Moore DM, Kalibala S. Factors associated with long-term antiretroviral therapy attrition among adolescents in rural Uganda: a retrospective study. J Int AIDS Soc. 2016 7 20;19(5 Suppl 4):20841 10.7448/IAS.19.5.20841 27443271 PMC4956735

[53] Kripke K, Njeuhmeli E, Samuelson J, Schnure M, Dalal S, Farley T, Hankins C, Thomas AG, Reed J, Stegman P, Bock N. Assessing progress, impact, and next steps in rolling out voluntary medical male circumcision for HIV prevention in 14 priority countries in eastern and southern Africa through 2014. PLoS ONE. 2016;11:e0158767 10.1371/journal.pone.0158767 27441648 PMC4955652

[54] Nanvubya A, Ssempiira J, Mpendo J, Ssetaala A, Nalutaaya A, Wambuzi M, Kitandwe P, Bagaya BS, Welsh S, Asiimwe S, Nielsen L, Makumbi F, Kiwanuka N. Use of modern family planning methods in fishing communities of Lake Victoria, Uganda. PLoS ONE. 2015;10:e0141531 10.1371/journal.pone.0141531 26512727 PMC4626115

[55] Borgdorff MW, Kwaro D, Obor D, Otieno G, Kamire V, Odongo F, Owuor P, et al HIV incidence in western Kenya during scale-up of antiretroviral therapy and voluntary medical male circumcision: a population-based cohort analysis. Lancet HIV. 2018;5;e241–e249 10.1016/S2352-3018(18)30025-0 29650451

[56] Rosenberg NE, Graybill LA, Wesevich A, McGrath N, Golin CE, Maman S, et al The impact of couple HIV testing and counseling on consistent condom use among pregnant women and their male partners: an observational study. J Acquir Immune Defic Syndr. 2017;75:417–425. 10.1097/QAI.0000000000001398 28426440 PMC5493523

[57] Fiorillo SP, Landman KZ, Tribble AC, Mtalo A, Itemba DK, Ostermann J, et al Changes in HIV risk behavior and seroincidence among clients presenting for repeat HIV counseling and testing in Moshi, Tanzania. AIDS Care. 2012;24:1264–1271. 10.1080/09540121.2012.658751 22375699 PMC3394906

[58] Kurth AE, Lally MA, Choko AT, Inwani IW, Fortenberry JD. HIV testing and linkage to services for youth. J Int AIDS Soc. 2015;18:19433 10.7448/IAS.18.2.19433 25724506 PMC4344538

[59] McMahon JH, Elliott JH, Bertagnolio S, Kubiak R, Jordan MR. Viral suppression after 12 months of antiretroviral therapy in low- and middle-income countries: a systematic review. Bull World Health Organ. 2013;91:377–385E. 10.2471/BLT.12.112946 23678201 PMC3646348

[60] Lungu A, Hüsken S. Assessment of access to health services and vulnerabilities of female fish traders in the Kafue Flats, Zambia In. Lusaka: The WorldFish Center; 2010.

[61] Ettarh RR, Kimani J. Influence of distance to health facilities on the use of skilled attendants at birth in Kenya. Health Care for Women Int. 2016;37:237–249. 10.1080/07399332.2014.908194 24730670

[62] Akullian A, Onyango M, Klein D, Odhiambo J, Bershteyn A. Geographic coverage of male circumcision in western Kenya. Medicine (Baltimore) 2017,96:e5885. 28079830 10.1097/MD.0000000000005885 PMC5266192

[63] Galbraith JS, Ochieng A, Mwalili S, Emusu D, Mwandi Z, Kim AA, et al Status of voluntary medical male circumcision in Kenya: findings from 2 nationally representative surveys in Kenya, 2007 and 2012. J Acquir Immune Defic Syndr. 2014;66 Suppl 1:S37–45. 24732820 10.1097/QAI.0000000000000121 PMC4794989

[64] Akullian A, Onyango M, Klein D, Odhiambo J, Bershteyn A. Geographic coverage of male circumcision in western Kenya. Medicine (Baltimore). 2017;96:e5885 10.1097/MD.0000000000005885 28079830 PMC5266192

[65] Odoyo-June E, Agot K, Grund JM, Onchiri F, Musingila P, Mboya E, Emusu D, Onyango J, Ohaga S, Soo L, Otieno-Nyunya B. Predictors of voluntary medical male circumcision prevalence among men aged 25–39 years in Nyanza region, Kenya: Results from the baseline survey of the TASCO study. PLoS ONE. 2017;12:e0185872 10.1371/journal.pone.0185872 28982175 PMC5628861

[66] Tenkorang EY. Marriage, widowhood, divorce and HIV risks among women in sub-Saharan Africa. Int Health. 2014 3;6(1):46–53. 10.1093/inthealth/ihu003 24480991

[67] de Walque D^1^, Kline R. The association between remarriage and HIV infection in 13 sub-Saharan African countries. Stud Fam Plann. 2012;43:1–10. 23185867 10.1111/j.1728-4465.2012.00297.x

[68] Agot KE, Vander Stoep A, Tracy M, Obare BA, Bukusi EA, Ndinya-Achola JO, Moses S, Weiss NS. Widow inheritance and HIV prevalence in Bondo District, Kenya: baseline results from a prospective cohort study. PLoS ONE. 2010;5:e14028 10.1371/journal.pone.0014028 21103347 PMC2984493

[69] Perry B, Oluoch L, Agot K, Taylor J, Onyango J, Ouma L, Otieno C, Wong C, Corneli A. Widow cleansing and inheritance among the Luo in Kenya: the need for additional women-centred HIV prevention options. J Int AIDS Soc. 2014;17:19010 10.7448/IAS.17.1.19010 24973041 PMC4074366

[70] Zaidi J, Grapsa E, Tanser F, Newell ML, Bärnighausen T. Dramatic increase in HIV prevalence after scale-up of antiretroviral treatment. AIDS. 2013;27:2301–5. 10.1097/QAD.0b013e328362e832 23669155 PMC4264533

[71] Smith DJ. Modern marriage, men's extramarital sex, and HIV risk in southeastern Nigeria. AmJ Public Health. 2007;97:997–1005. 17463366 10.2105/AJPH.2006.088583 PMC1874209

